# Partial Inhibition of Complex I Restores Mitochondrial Morphology and Mitochondria-ER Communication in Hippocampus of APP/PS1 Mice

**DOI:** 10.3390/cells12081111

**Published:** 2023-04-08

**Authors:** Jessica Panes, Thi Kim Oanh Nguyen, Huanyao Gao, Trace A. Christensen, Andrea Stojakovic, Sergey Trushin, Jeffrey L. Salisbury, Jorge Fuentealba, Eugenia Trushina

**Affiliations:** 1 Department of Neurology, Mayo Clinic, Rochester, MN 55905, USA; 2 Department of Physiology, Universidad de Concepcion, Concepción 4030000, Chile; 3 Department of Molecular Pharmacology and Experimental Therapeutics, Mayo Clinic, Rochester, MN 55905, USA; 4 Microscopy and Cell Analysis Core Facility, Mayo Clinic, Rochester, MN 55905, USA; 5 Department of Biochemistry and Molecular Biology, Mayo Clinic, Rochester, MN 55905, USA; 6 Centro de Investigaciones Avanzadas en Biomedicina (CIAB-UdeC), Universidad de Concepción, Concepción 4030000, Chile

**Keywords:** Alzheimer’s disease (AD), mitochondria, endoplasmic reticulum (ER), serial block-face scanning electron microscopy (SBFSEM), three-dimensional electron microscopy (3DEM), small molecule mitochondria targeted therapeutics

## Abstract

Alzheimer’s disease (AD) has no cure. Earlier, we showed that partial inhibition of mitochondrial complex I (MCI) with the small molecule CP2 induces an adaptive stress response, activating multiple neuroprotective mechanisms. Chronic treatment reduced inflammation, Aβ and pTau accumulation, improved synaptic and mitochondrial functions, and blocked neurodegeneration in symptomatic APP/PS1 mice, a translational model of AD. Here, using serial block-face scanning electron microscopy (SBFSEM) and three-dimensional (3D) EM reconstructions combined with Western blot analysis and next-generation RNA sequencing, we demonstrate that CP2 treatment also restores mitochondrial morphology and mitochondria-endoplasmic reticulum (ER) communication, reducing ER and unfolded protein response (UPR) stress in the APP/PS1 mouse brain. Using 3D EM volume reconstructions, we show that in the hippocampus of APP/PS1 mice, dendritic mitochondria primarily exist as mitochondria-on-a-string (MOAS). Compared to other morphological phenotypes, MOAS have extensive interaction with the ER membranes, forming multiple mitochondria-ER contact sites (MERCS) known to facilitate abnormal lipid and calcium homeostasis, accumulation of Aβ and pTau, abnormal mitochondrial dynamics, and apoptosis. CP2 treatment reduced MOAS formation, consistent with improved energy homeostasis in the brain, with concomitant reductions in MERCS, ER/UPR stress, and improved lipid homeostasis. These data provide novel information on the MOAS-ER interaction in AD and additional support for the further development of partial MCI inhibitors as a disease-modifying strategy for AD.

## 1. Introduction

Alzheimer’s disease (AD) is a devastating neurodegenerative disorder without a cure. The accumulation of misfolded amyloid beta (Aβ) peptides and hyperphosphorylated microtubule-associated tau protein (pTau) represents two hallmarks of AD. However, an incomplete understanding of the underlying molecular mechanisms hampers the development of disease-modifying strategies. The limited success of clinical trials focused on Aβ and pTau-reducing therapies emphasizes the need to identify new targets and therapeutic approaches. AD is a multifactorial disease that has developed over time, where neuroinflammation, altered energetics, and metabolism, together with genetic and environmental contributions, play important roles [1]. Abnormal energy homeostasis and mitochondrial dysfunction are among the earliest mechanisms of AD pathogenesis [2,3]. Reduced brain glucose metabolism is present in patients with mild cognitive impairment, a prodromal stage of AD, indicating that the mitochondrial ability to produce energy to maintain synaptic function may be compromised [4]. Indeed, reduced glucose uptake in the brain and low synaptic density better correlate with the development of cognitive abnormalities than levels of Aβ or pTau [4,5]. The origin of mitochondrial dysfunction in AD is not clear. Multiple studies demonstrated that Aβ and pTau could co-localize with mitochondria and disrupt their function, leading to increased production of reactive oxygen species (ROS), reduced ATP levels, and altered axonal trafficking that perturbs mitochondrial localization at the synapses and distal sites of axons [6,7,8]. Abnormal mitochondrial dynamics, including biogenesis (a process associated with the production of new organelles), fission, fusion, and the removal of damaged mitochondria via mitophagy, lead to the accumulation of dysfunctional organelles, increased ROS, and compromised cellular energetics [9]. However, emerging data also support the reciprocal relationship where mitochondria could affect APOE4, Aβ, and Tau homeostasis, raising the possibility that mitochondria could play underlying and contributing roles in AD development and progression [10,11,12,13,14,15,16]. Taken together, these findings support the notion that strategies to improve mitochondrial dynamics and function could be beneficial for AD.

Mitochondria, apart from producing energy to support synaptic transmission and cognitive function, also mitigate calcium (Ca^2+^) signaling, govern biosynthesis of macromolecules (e.g., lipids, heme, and iron-sulfur clusters), improve innate immunity, and induce apoptosis [17]. To maintain cellular and energy homeostasis, especially under stress conditions, mitochondria communicate with the nucleus and other cytoplasmic compartments by releasing various metabolites, ROS, peptides, and by changing mitochondrial membrane potential. Mitochondria directly interact with the endoplasmic reticulum (ER), forming close contacts (below 25 nm) known as the mitochondria-ER contact sites (MERCS, also biochemically known as mitochondria-associated membranes, MAMs) [18]. MERCS were originally identified in the 1950s using electron microscopy (EM) [19] and are recognized as platforms for the regulation of Ca^2+^ flux, lipid metabolism, autophagosome formation, ER stress, mitochondrial quality control, mitochondrial bioenergetics, and apoptosis [20]. Structural and molecular studies in AD reveal altered mitochondria-ER communication, a “hypergeneration” of MERCS, which facilitated the formation of Aβ, pTau, abnormal phospholipid and calcium homeostasis, mitochondrial dysfunction, and neurodegeneration [20,21]. MERCS are involved in the production of ceramides, core constituents of sphingolipids important for the regulation of cell growth, differentiation, cell-cycle arrest, cell survival, apoptosis, and inflammation [22]. Specifically, increased levels of ceramides Cer16, Cer18, Cer20, and Cer24 in the blood of AD patients were directly linked to oxidative stress and Aβ pathology [23,24]. 

Along with the exaggerated formation of MERCS in AD, changes in mitochondrial morphology were reported in model systems and postmortem AD brains [7]. Specifically, multiple studies demonstrated extensive mitochondrial fragmentation [25]. However, these findings were based on the application of transmission electron microscopy (TEM), which lacks complex dimensional architecture, or fluorescent microscopy with limited spatial resolution. In recent years, the development of advanced imaging techniques, such as serial block-face scanning electron microscopy (SBFSEM), has allowed three-dimensional (3D) assessment of mitochondrial structures in the context of the complex tissue architecture of the brain [26]. 3D reconstructions of consecutive brain slices generated using SBFSEM in postmortem brain tissue of AD patients and multiple mouse models of AD allowed us to identify a novel mitochondrial phenotype, mitochondria-on-a-string (MOAS) [7,27,28]. MOAS could exceed 5 μm in length and consist of multiple teardrop-shaped organelles connected with a thin double membrane of various length and thickness that are difficult to detect using methods other than 3D EM [28]. Other groups presented data showing that MOAS were prevalent in the brain of aged *Rhesus macaques* [29], in stressed striated muscles [30], and after transient global cerebral ischemia [31]. After reperfusion, mitochondrial morphology in ischemic brains was restored, indicating the potential reversibility of MOAS based on the reduced energetic stress [31]. The formation of MOAS was associated with fission arrest, where the first step of Drp1 translocation to mitochondria required for constriction was not affected but the final stages of fission appeared to be blocked [28,29,31]. The exact mechanism of fission delay and MOAS formation remains unclear. The relevance of MOAS to AD pathogenesis is not clear either, where, on the one hand, MOAS may be dysfunctional mitochondria undergoing active fission, where their formation could further exacerbate the AD progression. On the other hand, they may represent an intermediate, reversible phenotype that maintains residual mitochondrial function under stress conditions, ensuring neuronal survival [28]. The initiation of mitochondrial fission is facilitated by the smooth ER [32]. However, the interaction of MOAS with the ER has not been systematically investigated. No data exist describing to what extent MOAS formation affects MERCS. Until now, the complex structure of MERCS was also visualized using standard 2D TEM, lacking the clarity of 3D architecture. Thus, since mitochondria in AD undergo multiple morphological transformations, the understanding of the complexity of their dynamics and interaction with other organelles requires the application of advanced imaging techniques.

We recently identified a small molecule tricyclic pyrone compound (code name CP2) that penetrates the blood-brain barrier and accumulates in mitochondria, where it selectively and specifically reduces the activity of mitochondrial complex I (MCI) [33,34,35]. Our data demonstrate that at concentrations relevant to in vivo treatment efficacy, CP2 inhibits ~20% of complex I activity without induction of ROS or switching to glycolysis [33,34,35]. Safety of CP2 administration was confirmed in experiments where breeding wild-type mice were exposed to 50 mg/kg/day for 30 days. Comprehensive histopathological examination of adult and newborn mice confirmed the lack of toxicity or the effect on development [33]. Mice did not show any signs of toxicity after chronic CP2 administration for 14 months [34]. A genome-wide association study conducted on 196 lymphoblastoid cell lines from individuals of different sexes, ages, and races also confirmed the safety of CP2 treatment [36]. Chronic CP2 administration in independent cohorts of transgenic AD mice starting in utero and at pre- or symptomatic stages of the disease improved synaptic function, reduced levels of Aβ and pTau, decreased inflammation, and oxidative stress, ultimately blocking the ongoing neurodegeneration and cognitive dysfunction [33,34,35,37]. Targeted metabolomic profiling conducted in the plasma of CP2-treated female mice harboring familial AD mutations in human amyloid precursor protein (APP) and presenilin 1 (PS1), the APP/PS1 mice, revealed a reduction in levels of ceramides Cer16, Cer18, and Cer24 implicated in excessive MERCS formation [23], suggesting that treatment might also have improved mitochondria-ER communication [34]. Cross-validation of the RNA-seq data generated in female APP/PS1 mice treated with CP2 for 14 months against the human dataset available via the accelerating medicine partnership program for AD (AMP-AD) confirmed the translational value of the mouse model and demonstrated that CP2 treatment restored pathways essential for human AD, including inflammation, oxidative stress, and synaptic function [34]. 

Application of MCI inhibitors to treat AD appears counterintuitive since mitochondrial dysfunction is detected early in the disease’s progression. In addition, the inhibitors that strongly and irreversibly inhibit MCI activity, such as rotenone or 1-methyl-4-phenyl-1,2,3,6-tetrahydropyridine (MPTP), are employed to generate models of Parkinson’s disease (PD) [38]. However, the underlying role of MCI deficiency in PD patients is still debated [39,40,41]. Interestingly, most treatments aimed at boosting mitochondrial function or reducing the pathology associated with increased production of ROS have failed clinical trials [42,43]. Unexpectedly, genetic or pharmacological reduction in the activity of the complexes involved in the oxidative phosphorylation (OXPHOS) and electron transport chain (ETC) machinery has been shown to provide significant health benefits, improving mitochondrial function and cellular energetics in multiple model systems in vitro and in vivo [44]. A mechanistic explanation of such unexpected benefits is rooted in the concept of an adaptive stress response. For example, non-pharmacological approaches, such as diet and exercise, reduce major AD hallmarks by engaging an adaptive stress response that leads to an improved metabolic state, reduced oxidative stress and inflammation, and improved proteostasis [7,45,46]. In the case of partial MCI inhibitors, health benefits could be attributed to mitochondria-mediated signaling that includes interaction with subcellular organelles and the nucleus via releasing metabolites of the TCA cycle, ROS, changes in membrane potential, and fission/fusion dynamics [17,47]. The extensive evaluation of the molecular mechanisms induced by CP2 treatment suggests a multifaceted adaptive stress response with the activation of AMP-activated protein kinase (AMPK) in response to an increase in AMP/ATP levels [33]. Additional mechanisms include activation of autophagy, mitochondrial biogenesis and turnover, increased levels of Sirtuins 1 and 3, improved glucose uptake and utilization, energy homeostasis in the brain and periphery, and reduced oxidative stress and inflammation, leading to neuroprotection [34,37,44]. These findings support the notion that mild energetic stress could produce a beneficial effect by engaging hormetic responses similar to the effect of exercise and calorie restriction [44,45,48]. Our studies in AD mice, with ongoing neurodegeneration and abnormal mitochondrial function similar to that observed in AD patients, support the safety and efficacy of this innovative therapeutic strategy, where mild inhibition of MCI results in activation of multiple neuroprotective pathways without targeting them individually, and which mimics a polypharmacy approach for the treatment of complex neurodegenerative diseases. Here, using advanced SBFSEM and 3D EM reconstruction, Western blot, and RNA-seq data analyses of the brain tissue, we demonstrate that an adaptive stress response to partial inhibition of MCI results in improved mitochondrial morphology, reduced MERCS, and alleviated ER and unfolded protein response (UPR) stress in the hippocampus of APP/PS1 female mice, which additionally could contribute to neuroprotection.

## 2. Materials and Methods

### 2.1. Reagents and CP2 Synthesis

All reagents were purchased from Sigma-Aldrich unless indicated otherwise. Trump’s solution, consisting of 4% formaldehyde +0.1% glutaraldehyde in a 0.1 M phosphate buffer, was made monthly in-house and stored at 4 °C. CP2 was synthesized by the Nanosyn, Inc. biotech company as described previously [34] and purified using HPLC. Authentication was completed using NMR spectra to ensure the lack of batch-to-batch variation in purity. CP2 was synthesized as a free base. 

### 2.2. Mice and Chronic In Vivo CP2 Treatment

Brain tissue for all experiments described in this paper was obtained from the study reported here [34]. The following female mice were used: double transgenic APP/PS1 and their non-transgenic (NTG) littermates [46]. Genotypes were determined by PCR, as described in [49]. All animals were kept on a 12 h–12 h light-dark cycle with a regular feeding and cage-cleaning schedule. Mice were randomly selected for study groups based on their age and genotype. NTG and APP/PS1 female mice (*n* = 16–21 per group) were given either CP2 (25 mg/kg/day in 0.1% polyethylene glycol [PEG] dissolved in drinking water *ad lib*) or vehicle-containing water (0.1% PEG) starting at 9 months of age [33]. Mice were housed five per cage, and water consumption and weight were monitored weekly. CP2 concentration was adjusted weekly based on mouse weight/water consumption. Independent groups of mice were continuously treated for 14 months until the age of 23–24 months. After the beginning of CP2 treatment, mice were subjected to a battery of tests, including in vivo FDG-PET and blood-based metabolomics, as described in [34]. After mice were sacrificed, brain tissue was collected for Western blot analysis, RNA sequencing, and electron microscopy examination. 

### 2.3. Tissue Dissection for Electron Microscopy Examination

Non-sedated mice were sacrificed by cervical dislocation to avoid technical artifacts in mitochondrial morphology associated with perfusion fixation or the application of anesthetics [50]. The whole brain was quickly removed (<2 min), the hemispheres were separated, and one of the hemispheres was used for dissection of the CA1 region for the EM examination. The rest of the brain was cut and frozen for Western blot analysis and RNA sequencing. The hippocampal CA1 region was dissected from vehicle- and CP2-treated NTG and APP/PS1 mice, cut into 2 mm^3^ pieces, and immediately transferred to a glass-bottom dish containing 1% glutaraldehyde +4% paraformaldehyde in 0.1 M phosphate buffer (Trump’s fixative). Tissues were kept overnight at room temperature. The next day, tissues were placed in 0.1 M phosphate buffer and stored at 4 °C until processing for SBFSEM. 

### 2.4. Serial Block-Face Scanning Electron Microscopy (SBFSEM) and 3D EM Reconstruction

Serial images for 3D EM reconstructions were obtained using a Thermo Fisher VolumeScope 2 SEM, which combines an integrated microtome and a high-resolution field-emission scanning electron microscope (SEM) to image through a sample in the z-axis. Following a 24 h fixation, the tissues were prepared for SBFSEM using a protocol developed by the National Center for Microscopy and Imaging Research (La Jolla, CA) [51]: (1) samples were rinsed 4 × 3 min in 0.1 M cacodylate buffer +2 mM CaCl_2_, (2) incubated in 2% osmium tetroxide in 0.15 M cacodylate buffer for 1.5 h rotating at RT, (3) incubated in 2% osmium tetroxide +2% potassium ferrocyanide in 0.1 M cacodylate for 1.5 h rotating at RT, (4) rinsed in H_2_O 4 × 3 min, (5) incubated in 1% thiocarbohydrazide (TCH) for 45 min at 50 °C, (6) rinsed in H_2_O 4 × 3 min, (7) incubated in fresh 2% osmium tetroxide in H_2_O for 1.5 h rotating at RT, (8) rinsed in H_2_O 4 × 3 min, (9) incubated in 1% aqueous uranyl acetate overnight at 4 °C, (10) further incubated in uranyl acetate for 1 h at 50 °C, (11) rinsed in H_2_O 4 × 3 min, (12) incubated in lead aspartate for 1 h at 50 °C, (13) rinsed in H_2_O 4 × 3 min, (14) dehydrated through ethanol series (60, 70, 80, 95, 100, 100%) for 10 min each, (15) rinsed two times in 100% acetone for 10 min each, (16) resin 1:2, 1:1, 3:1 in acetone 0.5 h, 1 h, 2 h, overnight in 100% resin. Samples were embedded into Embed-812 hard resin (EMS, Hatfield, PA, USA), and allowed to polymerize at a minimum of 24 h prior to trimming and mounting. The tissue was trimmed of all surrounding resin and adhered to 8 mm aluminum pins (Ted Pella Inc., Redding, CA, USA) using EpoTek silver epoxy (EMS, Hatfield, PA, USA). A square tower (0.5 mm) was trimmed from the tissue using a Diatomeultratrim knife (EMS, Hatfield, PA, USA), and the entire pin was coated with gold palladium. Serial block-face images were acquired using a Thermo Fisher Volumescope 2 SEMTM (Thermo Fisher, Inc., Waltham, MA, USA) under high-vac/low water vapor conditions with a starting energy of 1.5 keV. A sectioning depth of 50 nm provided a final voxel size of 8 nm × 8 nm × 50 nm. Segmentation and three-dimensional analysis were performed using Reconstruct [52] and Amira 6.4 software (Thermo Fisher, Inc., Waltham, MA, USA).

### 2.5. Image Segmentation and Quantitative Morphometric Analysis Using 3D EM

Dendrites, mitochondria, ER, and MERCS were segmented manually by tracing 10–40 consecutive serial sections (0.09 µm thick) to generate 3D voxel segmentations. After 3D reconstruction was completed using Reconstruct software, traces were modified using an absolute intensity and maximal thresholding approach and exported in JPEG format into Amira software. The Brush and Lasso tools in Amira segmentation mode were used to select each object. If the borders of an object were incomplete, it was excluded. Multiple data sets were volume rendered simultaneously, and each cellular structure was assigned a different color during the segmentation process using 3D threshold-based selection. 

Quantitative analysis of mitochondrial morphology was conducted using the label analysis and measure tool functions in the Amira project view to obtain parameters related to mitochondrial shape (i.e., length, volume, aspect ratio, sphericity; Figures S1 and S2). The length was estimated using the ruler icon in the Amira Project view, based on the distance between any two most distant points in a 3D object. The volume was estimated using default measurements in Amira Native Measurements and measured in μm^3^. The aspect ratio (AR) was computed as [(major axis)/(minor axis)], which reflects the “length to width ratio”. The sphericity represents the adherence of the object to that of a sphere and was calculated using the equation:

$$\psi =\frac{\pi^{\frac{1}{3}}\times {(6V)}^{\frac{2}{3}}}{\mathrm{SA}}$$

where V refers to the volume of the object and SA refers to the surface area of the object, with a value of 1 indicating a perfect spheroid. The SA for each object was estimated automatically and measured using Amira Native parameters based on the total area of all mitochondrial faces.

The following classification for mitochondrial types observed in dendrites was adapted from [53]: Type I: round-shaped, no longer than 0.5 μm; Type II: medium-sized, with one mitochondrial tubule of 0.5 to 5 μm in length but no longer than 5 μm; Type III: elongated, with one mitochondrial tubule of 5 μm or longer; Type IV: mitochondria-on-a-string (MOAS), elongated, interconnected organelles with more than one teardrop-shaped mitochondria (~0.5 µm in diameter) connected by a thin double membrane extending up to 5 μm long (uniformly ~65 nm in diameter). The length and volume of MERCS were established using the measure tool in the Amira project view. The maximum distance between any segment of the ER and a mitochondrion was set at 25 nm to be considered a juxtaposed area of the mitochondria–ER interface. To measure the length of the contact, a freehand line was drawn from the beginning to the end of the mitochondrion-ER contact that was considered before. These values were then averaged to generate the length of contact sites for a given sample. To estimate mitochondrial coverage with MERCS, we calculated the percentage of mitochondrial perimeter covered by the area of individual MERCS (Figures S1 and S2). Representative images are included with the paper; additional images are available upon request directed to Dr. E. Trushina.

### 2.6. Mitochondrial Fractionation

This protocol was adapted from [54] with a few modifications. Protease and phosphatase inhibitors were added to the buffer to prevent protein degradation and dephosphorylation. A fresh mouse cortico-hippocampal region of the brain was washed with ice-cold phosphate buffer saline (PBS) to remove excess blood. The tissue was suspended in mitochondrial isolation buffer (MIBA) containing 10 mM Tris-HCl, pH 7.4, 1 mM EDTA, 0.2 M D-mannitol, 0.05 M sucrose, 0.5 mM sodium orthovanadate, 1 mM sodium fluoride, and 1× Complete Protease and Phosphatase Inhibitor cocktail ( Roche, Sigma-Aldrich, Burlington, MA, USA, cat. #1169749800), and homogenized with 10–20 strokes using a Teflon pestle (motor speed as 100). The crude nuclei (CN) fraction was pelleted from the lysate by centrifugation at 500× *g* at 4 °C for 5 min. The remaining supernatant was centrifuged at 500× *g* at 4 °C for 5 min to remove the micronuclei. Then, the supernatant was centrifuged at 8000× *g* for 10 min at 4 °C, yielding heavy mitochondrial (MT pellet) and cytoplasmic (CY supernatant) fractions. The MT pellet was washed twice with ice-cold MIBA buffer before it was resuspended in the lysis buffer. Three mice from each experimental group were taken for analysis.

### 2.7. Western Blot Analysis

Levels of proteins were determined in cortico-hippocampal regions of the brain or enriched mitochondria fractions from cortico-hippocampal brain regions obtained as described above from vehicle and CP2-treated NTG and APP/PS1 mice (*n* = 2–3 mice per group) using Western blot analysis. Samples were homogenized and lysed using 1× RIPA buffer plus inhibitors. Total protein lysates (20 µg) were separated in equal volumes on 4–20% Mini-PROTEAN TGX™ Precast Protein Gels (Bio-Rad, Hercules, CA, USA, cat. # 4561096) and transferred to a PVDF membrane (cat. # 1620177). The following primary antibodies were used: Drp1 (1:1000, BD Biosciences, San Jose, CA, USA), phospho-Drp1 S616 (1:1000, Cell Signaling, Danvers, MA, USA, cat. #3455), OPA1 (1:1000, Fisher Scientific, Waltham, MA, USA, cat. #BDB612607), Mfn1 (1:1000, EMD Millipore, Burlington, MA, USA cat. #ABC41-M), Mfn2 (1:1000, Sigma, Burlington, MA, USA, cat. # M6444-200UL), Vdac1 (1:1000, Proteintech, Rosemont, IL, cat. #55259-1-AP), Tfam (1:1000, Sigma-Aldrich, Burlington, MA, USA, cat. # AV36993), Tfeb (1:500, Thermo Fisher, Waltham, MA, USA, cat. # PA5-75572), LC3b (1:1000, Novus Biologicals, Centennial, CO, USA, cat. # NB100-2220), Pink1 (1:1000, Novus Biologicals, cat. #BC100-494), Parkin (PRK8) (1:1000, Santa Cruz, Santa Cruz, CA, USA, cat. #sc-32282), and β-Actin (1:5000, Sigma-Aldrich, Burlington, MA, USA, cat. # A5316). The following secondary antibodies were used: Peroxidase (HRP) Anti-Rabbit IgG (H+L) Goat Secondary Antibody (1:5000 dilution, Jackson ImmunoResearch, West Grove, PA, USA, cat. #111-035-003); and Peroxidase AffiniPure Goat Anti-Mouse IgG (H+L) (1:5000 dilution, Jackson ImmunoResearch, West Grove, PA, USA, cat. #115-035-003). Band quantification was performed using the ChemiDoc Imaging System from Bio-Rad. Data analysis was performed using Image J software.

### 2.8. Next-Generation RNA Sequencing

A detailed description of the next-generation RNA-seq data generated in cortico-hippocampal regions of the brain tissue from APP/PS1 and NTG mice treated with vehicle or CP2 for 14 months (*n* = 5 per group) has been published here [34]. Differential expression analysis was performed using the EdgeR package. For specific pathway analysis, we used a loose threshold of log2-fold-change ≥ 0.2 and *p*-values ≤ 0.05 as the cutoff. Barcode plots were generated using the limma package. RNA-seq data availability: https://www.ncbi.nlm.nih.gov/geo/query/acc.cgi?acc=GSE149248 (GEO accession ID is GSE149248). The code (R script) used to generate the final RNA-seq analysis is available here [34].

### 2.9. Statistics 

Statistical analyses were performed using the GraphPad Prism (Version 8, GraphPad Software, Inc., La Jolla, CA, USA). Statistical comparisons among the three groups were conducted by one-way ANOVA and the one-sided unpaired Student’s *t*-test, where appropriate. Significant differences between vehicle and CP2-treated groups within the same genotype and differences among NTG, APP/PS1, and APP/PS1 + CP2 mice were considered in the final analysis. Data are presented as: mean ± S.E.M. for each group of mice.

## 3. Results

### 3.1. Evaluation of Mitochondrial Morphology and MERCS in Brain Tissue of APP/PS1 and NTG Mice Using SBFSEM and 3D EM Reconstruction

**Figure 1:**
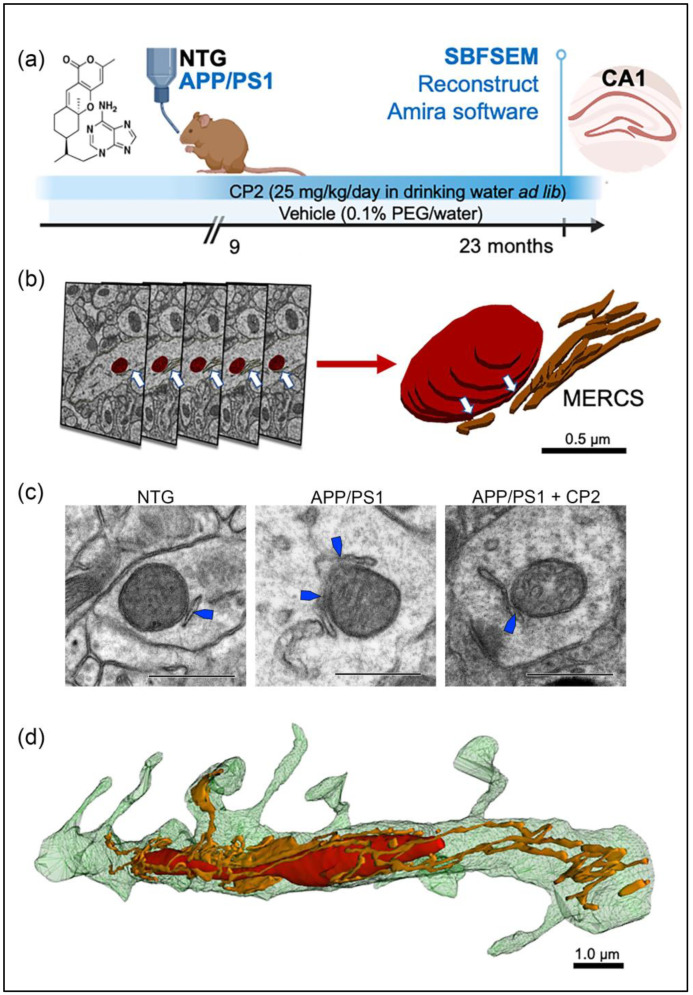
Treatment paradigm and workflow for 3D EM reconstruction of mitochondria and MERCS in brain tissue. (**a**) CP2 structure and a timeline of chronic CP2 administration to NTG and APP/PS1 female mice. (**b**) Serial block-face scanning electron microscopy (SBFSEM) with subsequent 3D EM reconstruction was executed in the CA1 hippocampal region using Reconstruct and Amira software. Mitochondria (red) and associated ER (white arrows) were traced in a dendrite through consecutive brain slices generated using SBFSEM (**left panels**). Resulting 3D EM reconstructions of mitochondria (red) and the ER (brown) allow to identify MERCS with high accuracy (white arrows). Scale bar, 0.5 µm. (**c**) Representative 2D TEM micrographs of MERCS (blue arrows) in NTG, APP/PS1 and APP/PS1+CP2 mice. Scale bar, 0.5 µm. (**d**) Representative 3D EM reconstruction of mitochondria (red) and the ER (gold) in a dendrite of a NTG mouse demonstrates the complexity of interaction. Reconstruction was performed using twenty-eight 0.09 μm thick TEM serial sections that were stacked, aligned, and reconstructed using Reconstruct software. Scale bar, 1 µm.

APP/PS1 female mice were treated with CP2 or vehicle starting at 9 months of age, after the onset of cognitive decline and neurodegeneration, to test the efficacy of this novel therapeutic strategy under conditions most relevant to patients (Figure 1a) [34]. NTG female mice were used as the control. After 14 months of chronic treatment, the CA1 hippocampal tissues were dissected for SBFSEM [50]. Cortico-hippocampal tissues from the same mice were dissected and used for Western blot, mitochondrial isolation, and RNA-seq analyses. The selection of the hippocampus for the evaluation was based on the fact that the CA1 region is affected early in AD, contributing to the loss of cognitive function [55]. Furthermore, we have shown that chronic CP2 treatment using the paradigm depicted in Figure 1a led to improved performance of APP/PS1 mice in cognitive tests, reduced amyloid plaques, restored mitochondrial morphology, dendritic spine morphology, and long-term potentiation in the hippocampus [34]. However, a detailed evaluation of the impact of CP2 treatment on ER stress, MERCS, or MOAS has not been performed. 

Compared to traditional 2D transmission EM (TEM, Figure 1c), 3D EM allows us to observe and quantify the morphological complexity of mitochondrial networks in great detail (Figure 1b,d) [56]. The example of the assessment of mitochondria-ER contacts using 2D TEM is presented in Figure 1c and Figure S3. While the differences between MERCS are clearly visible with increased mitochondria-ER interactions in APP/PS1 mice compared to NTG or APP/PS1 mice treated with CP2, the mitochondrial morphology (round, elongated, or MOAS) cannot be clearly distinguished. Similarly, the true extent of MERCS can only be revealed when assessing these interactions in 3D format (Figure 1d).

**Figure 2:**
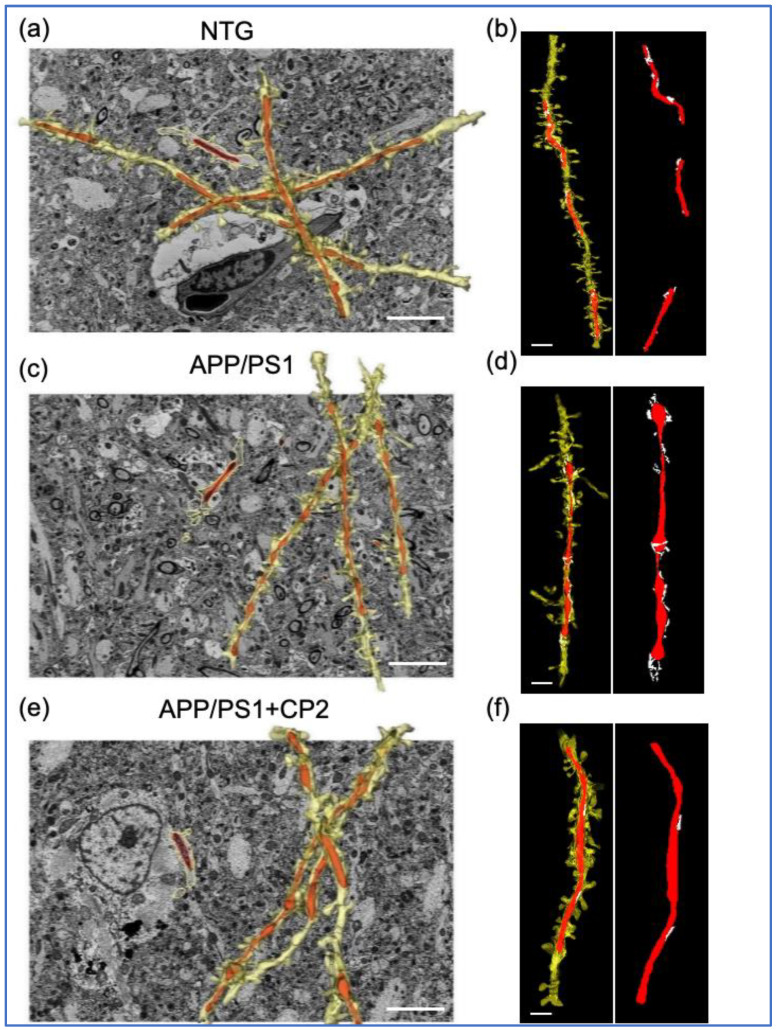
Application of SBFSEM and 3D EM reconstructions to establish changes in mitochondrial morphology and MERCS in response to CP2 treatment. Representative 3D EM reconstructions of dendrites (yellow) and mitochondria (red) in hippocampal CA1 region of NTG (**a**), APP/PS1 (**c**) and APP/PS1+CP2 (**e**) mice. Scale bars, 5 μm. Representative 3D EM reconstructions of dendrites (yellow), mitochondria (red), and the ER (white) in hippocampal CA1 region of NTG (**b**), APP/PS1 (**d**) and APP/PS1+CP2 (**f**) mice. Scale bars, 2 μm. The Reconstruct software was applied to micrographs from 20–40 serial sections 0.09 μm thick generated using SBFSEM.

For 3D EM reconstruction, 20–40 serial sections were generated using SBFSEM, stacked, aligned, and visualized using Amira (Figure 1b) or Reconstruct software (Figure 1d). This approach provides high quality samples and 3D visualization of mitochondria and ER morphology (Figure 1b–d). An advantage of 3D EM is the ability to observe and quantify the morphological complexity of mitochondrial networks in great detail [56]. Amira allows for segmentation, rendering, and color-coded visualization of 3D EM reconstructions to obtain quantitative data (Figures S1 and S2) [53,56]. Particularly, 3D volume segmentation provides clear identification of MERCS by visualizing contacts between mitochondria and the ER in 3D space. We limited volume reconstruction to dendrites since they could be unambiguously identified based on the presence of dendritic spines (Figure 1d and Figure 2). The precise identification of neuronal compartments occupied by mitochondria is another benefit of 3D EM compared to 2D TEM. 

Data were collected from NTG and APP/PS1 mice treated with vehicle or CP2 to determine the effect of treatment on mitochondrial shape, length, and the interaction with the ER (Figure 1 and Figure 2). The representative reconstructions of mitochondria and the ER (Figure 1b,c) in dendrites (Figure 1d and Figure 2) demonstrate the 3D architecture of mitochondria, including shape, volume, and the distance between mitochondria-ER membranes. Detailed descriptions of measurements using Amira software are presented in Figures S1 and S2.

### 3.2. CP2 Treatment Restores Mitochondrial Morphology in Symptomatic APP/PS1 Mice

**Figure 3:**
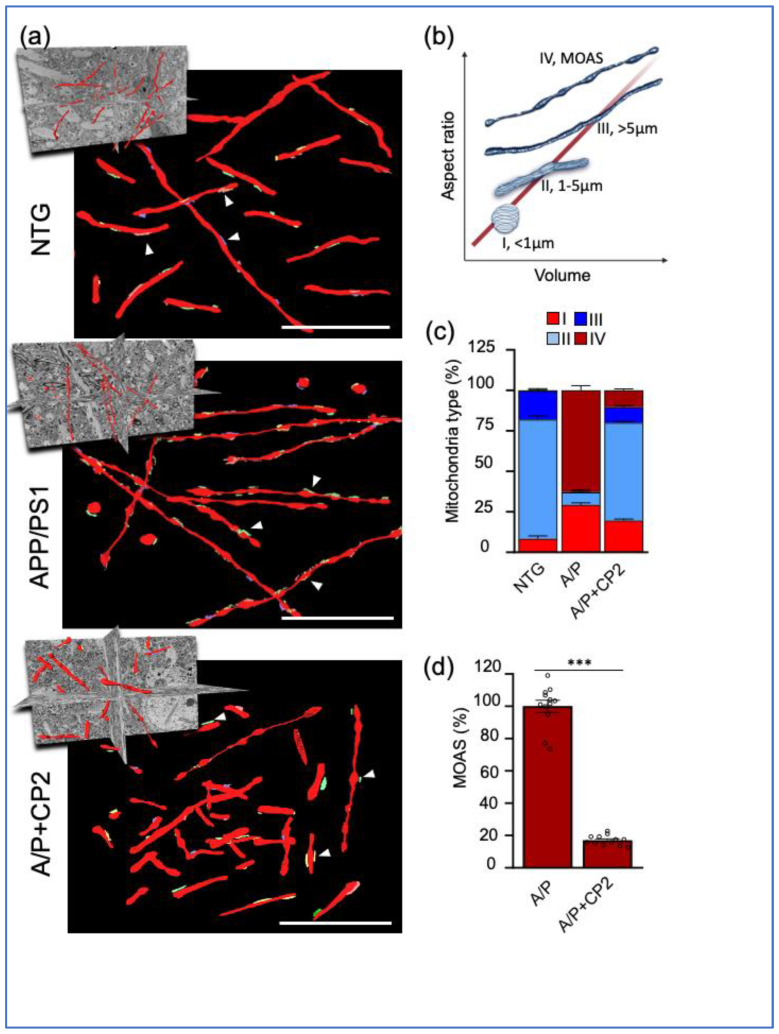
Chronic CP2 treatment improves mitochondrial morphology in APP/PS1 (A/P) mouse brain. (**a**) Visualization of mitochondrial morphology in the hippocampus of NTG, APP/PS1 and APP/PS1+CP2 mice using SBFSEM and 3D EM reconstructions using the Amira software. Mitochondria are in red; the ER membranes are in green, yellow, or blue. White arrows indicate representative MERCS. Scale bars, 5 μm. (**b**) Four morphological profiles were defined using mitochondrial aspect ratio and volume estimated using 3D EM reconstructions and the Amira software. (**c**) Quantification of mitochondrial Types I–IV in mouse brain. Morphometric analysis was conducted in a blind fashion using randomly selected 3D EM mitochondria reconstructions from NTG, APP/PS1 and APP/PS1+CP2 mice. Ten random sections with ~62–100 mitochondria were taken into the analysis for each group. (**d**) CP2 treatment reduces MOAS in hippocampus of APP/PS1 mice. Morphometric analysis was conducted as in (**d**). Unpaired Student’s *t*-test was used for all statistical analyses. Data are presented as mean ± SEM. *** *p* < 0.001.

We have previously shown that AD patients and multiple mouse models of AD, including APP/PS1 mice, have increased MOAS formation in the brain [28]. MOAS are formed in response to energetic stress associated with reduced glucose or oxygen availability [28]. We also found that CP2 treatment restored brain energy homeostasis in APP/PS1 mice [33,34,36]. Thus, we first examined the effect of CP2 treatment on mitochondrial morphology in APP/PS1 mice compared to NTG littermates (Figure 3). The 3D EM reconstructions of mitochondria were performed in randomly selected dendrites, with 60–100 organelles taken into the analysis per group (Figure 3a). To provide a quantitative assessment of mitochondrial morphology, mitochondria were classified into four distinct types based on their 3D aspect ratio (length vs. width) and volume (Figure 3b). Type I represents round mitochondria with a diameter below 1μm. Type II includes mitochondria with an elongated shape and a length of 1 to 5 μm. Type III consists of elongated organelles longer than 5 μm, and Type IV represents MOAS (Figure 3b). 

We found that in NTG mice, dendritic mitochondria in the hippocampus were predominantly of Type II (~70%), with a lower percent of Types I (~5%) and III (and ~15%) (Figure 3c). NTG mice did not have MOAS in the hippocampus. Consistent with our previous observations [28,34], vehicle-treated APP/PS1 mice had increased MOAS (up to 80% of total mitochondria) and Type I mitochondria (30% in APP/PS1 mice compared to 5% in NTG mice) (Figure 3c). We did not observe Type III mitochondria in APP/PS1 mice, while in NTG mice, this type represented ~15% of the total mitochondrial population. CP2 treatment restored the mitochondrial phenotype in APP/PS1 mice to that observed in NTG mice (Figure 3a–d). The most conspicuous was the reduction in MOAS by 80% (Figure 3d). We also found that CP2 treatment reduced the number of round-shaped Type I organelles and increased the number of Types II and III mitochondria in the brain tissue of CP2-treated APP/PS1 mice compared to their vehicle-treated counterparts (Figure 3c, Movie S1). These data confirmed that CP2 treatment not only improved brain energy homeostasis but also restored mitochondrial morphology, reducing fragmentation and MOAS formation in the hippocampus of APP/PS1 mice.

### 3.3. CP2 Treatment Reduces MERCS in APP/PS1 Mice

**Figure 4:**
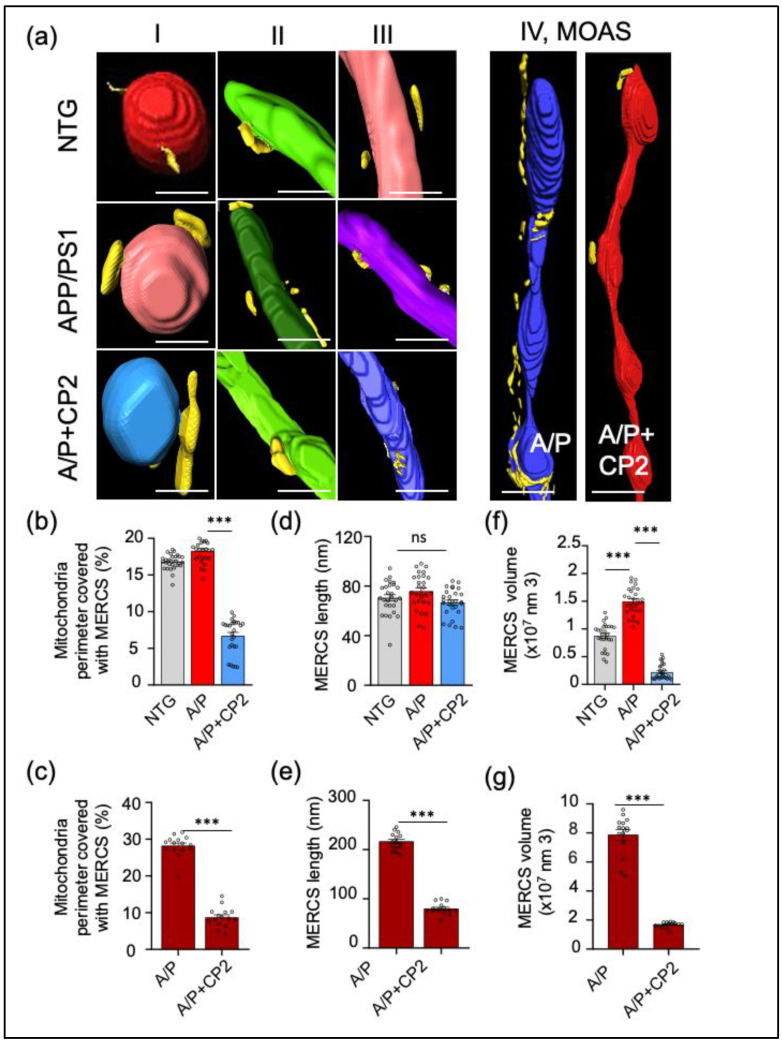
Chronic CP2 treatment reduces MERCS in the hippocampus of APP/PS1 (A/P) mice. (**a**) Representative 3D EM reconstructions of MERCS in NTG, APP/PS1, and APP/PS1+CP2 mice for randomly selected mitochondrial Types I–IV. Mitochondria are colored in red, blue, purple, green, and teal; the ER is yellow. Reconstruction was performed using SBFSEM images from the CA1 hippocampal region using the Amira software. MOAS from the hippocampus of an APP/PS1 mouse have exaggerated MERCS along the whole organelle. CP2 treatment decreases MERCS in the residual MOAS (**a**, **left panel**). Scale bars, 0.5μm. (**b**) Morphometric analyses show CP2 treatment significantly reduces MERCS coverage (**b**,**c**), length (**d**,**e**), and volume (**f**,**g**) for all Types of mitochondria and MOAS. Ten random sections with ~62–100 mitochondria were taken into the analysis for each group. One-way ANOVA and one-sided unpaired Student’s *t*-test were used for all statistical analyses. Data are presented as mean ± SEM. *** *p* < 0.001. ns, not significant.

Extensive MERCS formation in AD was shown to facilitate Aβ production, abnormal lipid homeostasis, ER stress, and apoptosis [57,58,59]. We previously reported that CP2 treatment reduced levels of Aβ in the hippocampus and blood levels of ceramides and blocked the ongoing neurodegeneration in APP/PS1 mice, indicating a potential improvement of mitochondria-ER communication and a reduction in ER and UPR stress [34]. To evaluate the relationship between different types of mitochondrial morphology and the ER, we utilized SBFSEM, 3D EM reconstruction, and the Amira software to visualize and quantify MERCS architecture in the hippocampus of NTG and APP/PS1 mice treated with vehicle or CP2 (Figure 4, Movie S2). MERCS were defined as the ER segments within a 25 nm or less proximity to mitochondria (Figure 1b and Figure 4a, Figures S1–S3). We estimated MERCS length, volume, and the percentage of mitochondrial surface covered by the ER. The analysis was conducted for mitochondrial Types I–III pooled together, while MOAS were analyzed separately. The 3D EM reconstructions reveal profound differences in the extent of mitochondria-ER interactions. For Types I–III, the presence of the ER membranes along the mitochondria was sporadic without the formation of a continuous network (Figure 4a). However, MOAS extensively interacted with the ER membranes (Figure 4a). The extent of mitochondrial Types I–III perimeter covered with the ER was similar in NTG and APP/PS1 mice (~18%, Figure 4b), while MOAS were covered to ~30% (Figure 4c). MERCS length did not differ significantly for Types I–III in NTG and APP/PS1 mice (~80 nm, Figure 4d), while the mean MERCS length for MOAS was ~200 nm (Figure 4e). The volume of MERCS associated with Types I–III mitochondria was significantly greater in APP/PS1 mice compared to NTG mice (Figure 4f) and was even greater for MOAS (Figure 4g). CP2 treatment reduced MERCS and their volume for all types of mitochondria in APP/PS1 mice (Figure 4b,c,f,g), and specifically reduced MERCS length associated with MOAS (Figure 4e). Taken together, these data further support the translational value of the APP/PS1 mouse model, demonstrating increased MERCS in the hippocampus, consistent with the presence of ER stress in AD. Furthermore, we found that, compared to all mitochondrial morphological phenotypes, the interaction with the ER was specifically prominent in MOAS. CP2 treatment not only restored mitochondrial morphology, decreasing MOAS in APP/PS1 mice, but also reduced MERCS for all types of mitochondria.

### 3.4. CP2 Treatment Augmented Mitochondrial Biogenesis and Turnover and Reduced ER Stress in APP/PS1 Mice

**Figure 5:**
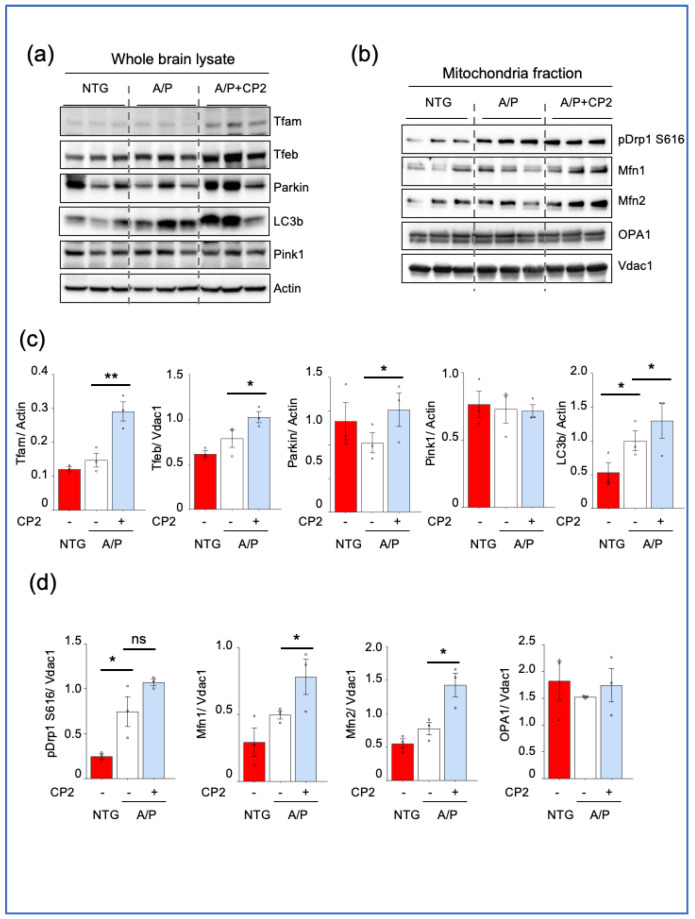
CP2 treatment enhances mitochondrial biogenesis and turnover in APP/PS1 (A/P) mice. Western blot analysis of protein expression in the cortico–hippocampal brain tissue lysates (**a**) or mitochondrial fractions (**b**) isolated from the cortico–hippocampal tissue of NTG, APP/PS1, and APP/PS1+CP2 mice. (**c**,**d**) Quantification of Western blots from (**a**,**b**), respectively. Differences between individual groups were analyzed using a one–sided, unpaired Student’s *t*-test. *n* = 3 mice per group. Data are presented as mean ± SEM. * *p* < 0.05, ** *p* < 0.005. ns, not significant.

The significant reduction in MOAS after CP2 treatment in APP/PS1 mice could be associated with improved glucose uptake and utilization in the brain, detected using in vivo 18F-fluorodeoxyglucose-positron emission tomography (FDG-PET) [28,34]. However, it remains unclear whether MOAS reduction was also associated with changes in mitochondrial turnover, including enhanced fission, fusion, biogenesis, and mitophagy. To address this question, we first conducted a Western blot analysis using extracts from cortico-hippocampal regions of the brain of NTG and APP/PS1 mice treated with vehicle or CP2 (Figure 5a,c and Figure S4). Brain tissues were from the same cohort of mice used for 3D EM reconstructions. We found that CP2 treatment increased levels of mitochondrial transcription factor A (Tfam), a master regulator of mitochondrial biogenesis. We next determined whether CP2 treatment enhanced mechanisms responsible for the removal of damaged organelles, which primarily occurs via autophagic degradation known as mitophagy. Indeed, levels of proteins essential for autophagy and mitophagy, including transcription factor EB (Tfeb), Parkin, and LC3b, were elevated after CP2 treatment in APP/PS1 mice (Figure 5a,c). Levels of Pink1 remained unchanged. 

We further assessed the effect of CP2 treatment on the expression of mitochondrial fission and fusion proteins essential for mitochondrial turnover. Our earlier studies revealed that the most effective way to determine changes in these proteins was to assay mitochondrial fractions isolated from the brain regions of interest [27,28]. Indeed, no changes in fission and fusion proteins were detected in the brain extracts from cortico-hippocampal areas (Figure S5). However, consistent with our previous findings showing increased translocation of the fission protein Drp1 to MOAS, we found an increase in the expression of Drp1 phosphorylated at S616 in extracts from mitochondria isolated from cortico-hippocampal tissue of APP/PS1 mice compared to NTG mice (Figure 5b,d, and Figure S6). CP2 treatment did not decrease levels of Drp1 S616 associated with mitochondria in APP/PS1 mice and showed a trend toward an additional increase. At the same time, we found increased levels of the fusion proteins Mfn1 and Mfn2 (Figure 5b,d). No changes were detected in levels of the fusion protein OPA1. These data indicate that the reduction in MOAS after CP2 treatment in APP/PS1 mice was associated with enhanced mitochondrial dynamics, including augmented fission, fusion, biogenesis, and mitophagy.

**Figure 6:**
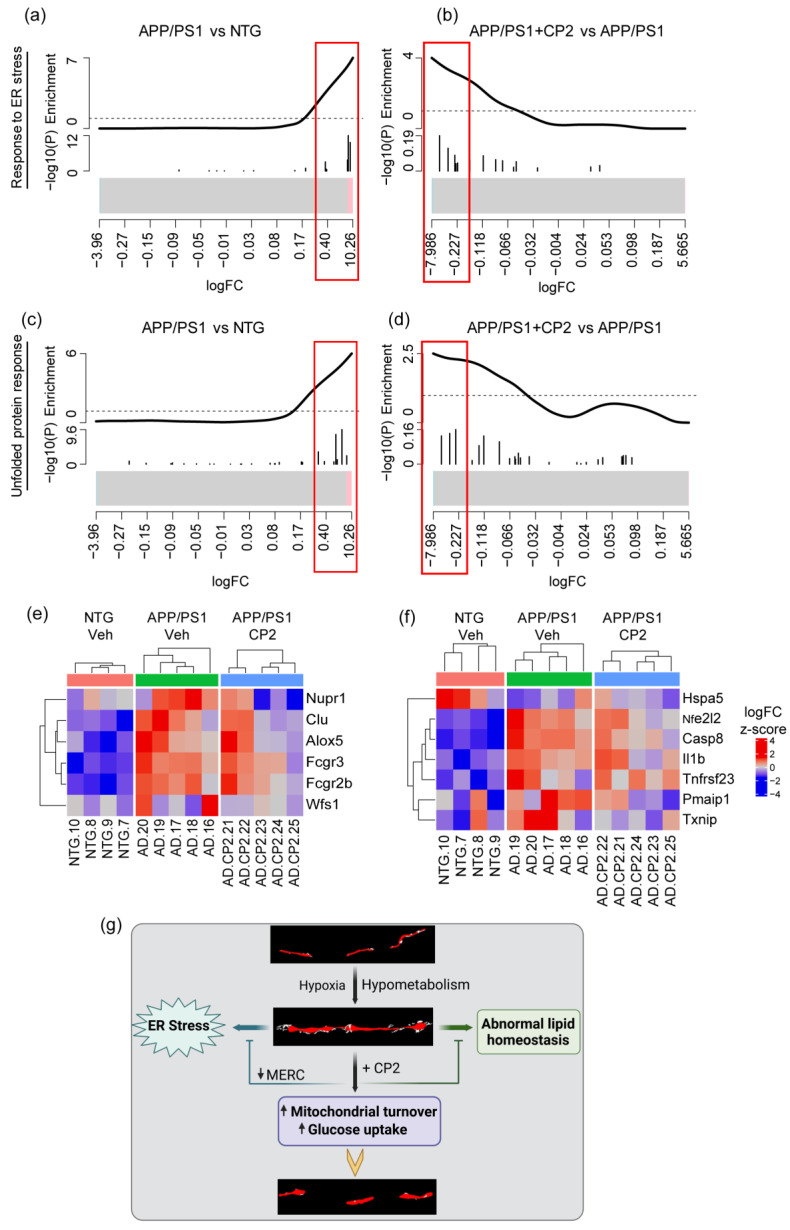
CP2 treatment reduces UPR and ER stress in APP/PS1 mice. Barcode plots (**a**–**d**) and heatmaps (**e**,**f**) show upregulated genes involved in ER stress (GO:1905897) (**a**,**b**,**e**) and UPR (WikiPathway:WP4925) (**c**,**d**,**f**) pathways in APP/PS1 mice that were down-regulated after CP2 treatment. Plots were generated by enrichment analysis using RNA-seq data from cortico-hippocampal brain tissue of APP/PS1 and NTG mice. (**a**–**d**) Barcode plots of ER stress pathway in APP/PS1 vs. NTG (**a**) and APP/PS1+CP2 vs. APP/PS1 (**b**), and the UPR pathway of APP/PS1 vs. NTG (**c**) and APP/PS1+CP2 vs. APP/PS1 (**d**). Genes that belong to specific pathways were marked by vertical lines in log2-fold-change scale, and enrichment was weighted by −log10 (*p*-values) from differential expression analysis between APP/PS1 and NTG or between APP/PS1+CP2 and APP/PS1. Red boxes indicate genes in core enrichment. (**e**,**f**) Heatmaps of significantly increased genes in ER stress pathway (**e**) and UPR pathway (**f**) that were reversed by CP2 treatment. Genes are labeled on the right; samples are labeled at the bottom and grouped by genotype/treatment. Expression for each gene was z-scored; color indicates z-score. (**g**) Schematic diagram of the relationship between mitochondrial morphology and early dysfunctions involved in AD, and the reversal by CP2 treatment.

To further investigate the mechanism of CP2 action, we utilized the RNA-seq data previously generated using cortico-hippocampal brain tissue from vehicle- and CP2-treated NTG and APP/PS1 mice from the same cohorts that we used in the current study [34]. We identified 27 differentially expressed genes (DEGs) involved in the UPR, ER stress, mitochondria-ER tethering, mitochondrial dynamics, and autophagy (Figure 6, Table S1). Specifically, we found upregulation of genes involved in the ER stress response mechanisms in APP/PS1 mice compared to NTG mice that were downregulated after CP2 treatment (Figure 6a,b,e). These genes included the nuclear transcriptional regulator protein 1 (*Nupr1*) involved in the regulation of autophagy-induced apoptosis through FOXO3 interaction, UPR, and the integrated stress response [60,61]. Nupr1 has been recently identified as a stress-induced transcription factor essential for blocking ferroptotic cell death associated with AD through diminishing iron accumulation and subsequent oxidative damage [62,63]. Another gene whose expression was reversed by CP2 treatment in APP/PS1 mice included clusterin (*Clu*). The gene product of *Clu* functions as an extracellular chaperone that prevents aggregation of proteins [64] and inhibits the formation of amyloid fibrils [65]. Moreover, clusterin interacts with ubiquitin and SCF (SKP1-CUL1-F-box protein) E3 ubiquitin-protein ligase complexes and promotes the ubiquitination and subsequent proteasomal degradation of target proteins [66]. Interestingly, mitochondrial clusterin suppresses BAX-dependent release of cytochrome c into the cytoplasm and inhibits apoptosis [67]. An intracellular form of clusterin suppresses stress-induced apoptosis by stabilizing mitochondrial membrane integrity through interaction with heat shock proteins Hspa5 [68]. Other genes included arachidonate 5-lipoxygenase (*Alox5*), Fc gamma receptor IIIa (*Fcgr3*), Fc gamma receptor IIb (*Fcgr2b*), and wolframin ER transmembrane glycoprotein (*Wfs1*), which are involved in mediating the inflammatory response, dendritic cell migration, apoptosis, and intracellular calcium signaling [69,70,71,72]. The Wfs1 gene product is directly involved in the regulation of cellular calcium homeostasis by modulating the ER calcium store [73]. 

Related to ER stress, another group of genes whose expression was reversed by CP2 treatment in APP/PS1 mice included the UPR pathway (Figure 6c,d,f). The expression of *Nfe2l2*, *Casp8*, *Il1b*, *Tnfrsf23*, *Pmaip1*, and *Txnip* was increased in APP/PS1 mice and reduced after CP2 treatment, while the expression of Hspa5 was reduced in APP/PS1 mice and increased after CP2 treatment (Figure 6f). The Hspa5 protein localizes to the lumen of the ER, where it is involved in the folding and assembly of the ER proteins, serving as a master regulator of the ER homeostasis. During cellular stress, Hspa5 interacts with the transmembrane stress sensor protein kinase R-like endoplasmic reticulum kinase (Perk), inositol-requiring kinase 1 (Ire1), and activating transcription factor 6 (Atf6), acting as a repressor of the UPR. Hspa5 also plays a role in cellular apoptosis and senescence [74], and CP2 treatment has been shown to reduce senescence and block neurodegeneration in APP/PS1 mice [34]. Other genes positively affected by CP2 treatment are involved in anti-apoptotic, anti-inflammatory, and antioxidant responses, including the master regulator of the oxidative stress response nuclear factor erythroid 2-related factor 2 (*Nfe2l2*), caspase 8 (*Casp8*), interleukin 1 beta (*Il1b*), tumor necrosis factor receptor superfamily, member 23 (*Tnfrsf23*), phorbol-12-myristate-13-acetate-induced protein 1 (*Pmaip1*), and thioredoxin interacting protein (*Txnip*) (Figure 6f). Importantly, Txnip has been recently identified as a link between redox state and metabolism, where it plays an essential role in normal glucose homeostasis [75]. Consistent with our observations of increased MERCS in APP/PS1 mice compared to NTG mice, we found significant upregulation of the expression of *Ripk1*, *Rrbp1*, *Sigmar1*, and *Tgm2* genes that play important roles at MERCS [76,77,78,79]. CP2 treatment reversed trends in the expression of these genes (Table S1). We also found that CP2 reversed changes in the expression of mitochondrial biogenesis regulator PGC-1α (*Ppargc1a*), mitochondrial dynamic protein MFN1 (*Mfn1*), and mitophagy-related protein Pink1 (*Pink1*), consistent with the Western blot analysis (Figure 5, Table S1). Collectively, these data suggest that CP2 treatment not only improves mitochondrial dynamics and reduces MOAS formation in APP/PS1 mice but also alleviates MERCS dysregulation, ER stress, and UPR stress.

## 4. Discussion

AD is a complex disorder, with multiple pathways affected during the disease’s development. These include inflammation, dysfunction of mitochondrial dynamics, function, and bioenergetics, oxidative stress, altered protein and Ca^2+^ homeostasis, and ER stress, among others. Since no biomarkers are available to clearly define at what stage of the disease what mechanism becomes affected, a successful approach to treatment may require targeting multiple pathways at the same time. Such a polypharmacy approach is very difficult to implement given the complicated etiology of AD. However, non-pharmacological approaches, such as exercise, have been shown to improve cognitive function, reduce inflammation and oxidative stress, and positively affect other major mechanisms underlying AD [48]. We identified MCI as a small-molecule druggable target for AD [33,34,36,37,44]. Similar to exercise, the partial inhibition of MCI induced an adaptive stress response and the activation of multiple mechanisms, each individually shown as neuroprotective [44]. The safety of this approach was tested in multiple experiments where transgenic AD mice were chronically treated through life [33,36] or for 14 months after the development of AD-like symptoms, as in this study [34]. We consistently observed the lack of toxicity and the improvement of motor and cognitive functions, suggesting that this treatment did not cause any adverse side effects or PD-like symptoms. Our data are consistent with multiple studies demonstrating that chronic application of metformin, another MCI inhibitor that is an FDA-approved drug to treat diabetes, is generally safe in the aging population. Metformin has been shown to promote healthy aging and alleviate neurodegeneration without inducing Parkinsonism [80,81,82]. Moreover, metformin is administered at much higher concentrations: mM compared to nM in the case of CP2. These data provide further support for the feasibility of chronic administration of MCI inhibitors without detrimental side effects. Most relevant to patients’ situations was the demonstration that CP2 treatment, starting after the onset of cognitive decline and neurodegeneration, was efficacious in restoring key disease mechanisms, including abnormal energy homeostasis, synaptic dysfunction, inflammation, abnormal lipid homeostasis, and accumulation of Aβ and pTau [34,37]. Among the most important observations were the improvement of mitochondrial morphology, dynamics, and function; restoration of axonal trafficking; and increased levels of neuroprotective mitochondrial Sirtuin 3 that collectively contributed to the halt of the ongoing neurodegeneration in APP/PS1 mice [34,83,84]. These findings suggested that CP2 worked even in cells where mitochondrial function was already diminished by the disease. It is feasible to assume that even in cells affected by AD, mitochondria range from completely functional to those that have various degrees of dysfunction. Currently, it is unclear whether efficacious treatment with MCI inhibitors requires a certain threshold of functional mitochondria and residual activity of complex I. This question is under active investigation in our laboratory. From this perspective, the examination of the effect of CP2 on mitochondria with different morphological phenotypes associated with AD, MOAS in particular, provides important information supporting the efficacy of treatment. Here, we followed up with the in-depth evaluation of the effect of CP2 on mitochondria-ER communication, MERCS, and mitochondrial morphology using SBFSEM and 3D EM reconstructions, the advanced imaging techniques with high resolution. 

One of the novel observations generated in this study was the demonstration that MOAS are increasingly associated with the ER, forming extensive MERCS. Until now, this phenomenon has not been described in the context of AD. Our data suggest that MOAS, a mitochondrial phenotype formed in response to energetic stress, has extensive interactions with the ER, greater than any other type of mitochondria. Currently, the precise mechanism of MOAS formation is not well understood, and it is unclear why MOAS are increasingly associated with the ER membranes. However, given the important role the ER plays in mitochondrial dynamics, fission in particular, such interaction may be instrumental for MOAS formation, promoting fission arrest under conditions of energetic stress, as well as MOAS elimination after stress relief [85]. Our previous studies showed that MOAS formation was not associated with the lack of Drp1 recruitment and self-assembly on mitochondria but with the GTP-driven constriction of the mitochondrial outer membrane [28]. Indeed, our data demonstrated that CP2 treatment improved energy homeostasis in the brain, leading to a significant reduction in MOAS that was not associated with increased recruitment of pDrp1 S616 to mitochondria. These data suggest that energy restitution may be sufficient to resume MOAS fission by Drp1, already assembled at MOAS constriction sites. Interestingly, the residual MOAS found in the brain of APP/PS1 mice after CP2 treatment had significantly less interaction with the ER, which may indicate that these structures were captured during their transition to different mitochondrial morphological types via resumed fission. In our previous study [28], we proposed that MOAS formation could be beneficial for supporting residual mitochondrial activity, which would have been impaired if organelles were divided and eliminated via mitophagy. However, few other publications that described MOAS formation in the aging brain [29] and under hypoxic conditions suggested that MOAS represent an ongoing fission that could be reversed after reperfusion [31,86]. A more thorough understanding of the molecular mechanisms of MOAS formation and reversal in real time in different brain regions remains to be determined experimentally. Nonetheless, the studies presented here suggest that MERCS play an important role in MOAS formation by either contributing to fission or facilitating fission arrest. 

Partial restoration of brain energy homeostasis after CP2 treatment reduced MOAS formation and their interaction with the ER. CP2 treatment also reduced the number of small mitochondria, whose formation is commonly associated with mitochondrial dysfunction in AD [12]. Most of the mitochondria in the CP2-treated APP/PS1 mouse brain were uniformly elongated, indicating increased fusion, consistent with our previous observations [27,33,34,36]. Indeed, Western blot and RNA-seq data analyses indicate that treatment enhanced mitochondrial turnover by restoring organelles and removing the damaged ones. The increased organellar fusion, which is associated with improved function, is also consistent with reduced oxidative stress detected after prolonged CP2 treatment [34,37], enhanced bioenergetics, and improved ATP production in the brain of CP2-treated APP/PS1 mice detected in vivo using ^31^P Nuclear Magnetic Resonance (^31^P NMR) Spectroscopy and in primary mouse neurons in vitro [34]. These data suggest a connection between abnormal glucose uptake and utilization in AD that results in increased MOAS and MERCS formation, ER stress, and abnormal lipid homeostasis, consistent with the identification of these mechanisms as the underlying cause of AD etiology (Figure 6g).

Our findings further demonstrate that CP2 treatment may not only be beneficial for AD but also improve health span, delaying the onset of AD in aging individuals. We demonstrated that the mitochondrial MERCS coverage was reduced by CP2 treatment in APP/PS1 mice to a level below that observed in age-matched 24-month-old NTG mice. Since increased MERCS were found to be associated with senescence and aging [87], this may indicate the potential beneficial effect of treatment also in aged NTG mice. However, in this work, we did not address the effect of CP2 treatment on MERCS and MOAS in aged NTG mice. We previously reported that 24-month-old NTG mice chronically treated with CP2 for 14 months did not show any signs of toxicity or side effects. Aged CP2-treated NTG mice demonstrated improvements in motor and cognitive functions, a reduction in inflammation and oxidative stress, and improved mitochondrial dynamics and glucose homeostasis [34]. We also found that CP2 treatment reduced levels of senescent cells in adipose tissue of CP2-treated APP/PS1 and NTG mice [34]. These data are consistent with the findings showing the MCI as a small-molecule-sensitive modifier of lifespan, where treatment of short-lived killifish with very low doses (pM) of the MCI inhibitor rotenone reversed aging-related changes in gene expression and extended lifespan [88]. Similarly, metformin, an MCI inhibitor, and resveratrol, an inhibitor of complexes I, III, and V [89], have well-known positive effects on health and life span [80,90,91]. Nevertheless, further investigation is needed to evaluate the rejuvenating effect of CP2 treatment, including mitochondria-ER communication.

Another important outcome of this study is the validation of the translational value of APP/PS1 mice that recapitulate changes in mitochondrial morphology and MERCS hypergeneration found in AD patients [28,92]. Many promising therapeutic strategies shown to be efficacious in mouse models of AD failed clinical trials, emphasizing the need for better preclinical models. APP/PS1 mice recapitulate many AD pathologies, providing confidence in the translational success of this novel treatment. APP/PS1 mice have increased levels of blood ceramides associated with abnormal mitochondria-ER communication similar to that found in the blood of AD patients [34]. CP2 treatment reduced levels of ceramides and MERCS, indicating that therapeutic efficacy could be monitored using blood-based translational biomarkers. This reduction in MECRS in APP/PS1 mice by CP2 treatment may also restore Ca^2+^ signaling, which also might have contributed to the increased neuronal survival and cognitive protection [34]. Transcriptomic data generated in the brains of APP/PS1 mice overlapped with changes in AD patients [34]. Here, we re-analyzed these data and demonstrated the presence of UPR and ER stress, which was reduced by CP2 treatment, further supporting the translational value of this mouse model. ER stress is commonly defined as a response to the build-up of unfolded or misfolded proteins within the ER, affecting the ER and cellular homeostasis [93]. Recent data also suggest the reciprocal relationship between abnormal mitochondria-ER communication and ER stress, which contributes to the onset and development of AD. Given the significance of the impact of ER stress on AD progression, multiple therapeutic approaches have been developed to mitigate ER stress, with only a few being tested in clinical trials [93]. Our data indicate that, without directly targeting the UPR or ER stress mechanisms, CP2 treatment reduced both. The CP2-dependent reversal of the expression of genes involved in ER/UPR stress and mitochondria-ER tethering reported in this and our previous study [34] further supports the relevance of this therapeutic strategy to patients. Among disease-related changes recently identified in a large-scale, deep multi-layer proteomic analysis of AD brain, fidelity of pathways involved in glycosylation/ER and post-synaptic density were associated with milder pathological symptoms and better cognitive function [8]. 

Restored MERCS and mitochondrial morphology, improved lipid and glucose homeostasis, and reduced UPR and ER stress in APP/PS1 mice after CP2 treatment coincide with reductions of Aβ and pTau [26,28]. The hierarchy of the beneficial mechanisms activated by CP2 treatment remains to be established. Our current findings suggest that activation of AMPK may play an essential role in the beneficial mechanistic cascade, similarly to that identified for metformin and resveratrol [33,34,36,37,44,81,94]. AMPK is a master regulator of cellular energy homeostasis that controls multiple mechanisms essential for neuronal survival and function. These mechanisms include synaptic transmission [95], mitochondrial biogenesis and turnover [96,97], inflammation [98,99], and the dynamics of MERCS [100], among others. However, excessive AMPK activation could be detrimental in AD [101]. Thus, future studies are needed to address the safety and efficacy of targeting MCI for AD treatment. In this work, we focused exclusively on dendrites. Thus, it remains to be determined whether similar beneficial effects on mitochondrial morphology and MERCS are elicited by CP2 treatment in axons and other brain cells. It will also be interesting to examine the effect of disease and CP2 treatment on synaptic mitochondria. In conclusion, using advanced imaging techniques, we provide new evidence that partial inhibition of MCI with the small molecule CP2 improves mitochondrial dynamics and mitochondria-ER communication in dendrites in the hippocampus of APP/PS1 mice and reduces UPR and ER stress. These data further support the relevance of this strategy for AD.

## Data Availability

RNA-seq data is available from Gene Expression Omnibus (GEO accession ID is GSE149248). The code (R script) used to generate the final RNA-seq analysis is available in Supplementary Data 22, 23 published here [34].

## Supplementary Materials

The following supporting information can be downloaded at: https://www.mdpi.com/article/10.3390/cells12081111/s1, Figure S1. Three-dimensional segmentation using Amira software. Protocol for generating surfaces and representative ortho slices of mitochondria-ER and MERCS, creating a video maker option for better visualization using Amira protocols. Figure S2. Three-dimensional segmentation in Amira for MERCS identification. (a) Segmentation and 3D rendering of ER (yellow), mitochondria (b, green), and Mito-ER segmentation (c) to generate MERCS surfaces (d, pink). (e) Measuring MERCS morphology using label analysis measurements to determine volume, length, and MERCS coverage per mitochondrial perimeter. Figure S3. Representative TEM of MERCS (blue arrows) in the hippocampus of NTG (a–d), APP/PS1 (e–h) and APP/PS1 + CP2 (i–l) mice. MOAS mitochondria are shown in (g) and (h). Scale bar, 0.5 μm. Figure S4. Original uncropped Western blot images for Figure 5a. Cortico-hippocampal regions from three mice per group were taken for Western blot analysis (NTG, *n* = 3; APP/PS1, *n* = 3; APP/PS1 + CP2, *n* = 3). Figure S5. (a) Expression of key fission/fusion proteins in cortico-hippocampal brain extracts from NTG and APP/PS1 mice treated with vehicle or CP2. (b) Densitometry analysis of protein expression from (a) normalized to actin. Differences between individual groups were analyzed by a one-sided unpaired Student’s t-test. Data are presented as the mean ± SEM from 2 to 3 mice. * *p* < 0.05; ** *p* < 0.005. Figure S6. Original, uncropped Western blot images for Figure 5b. Mitochondrial fractions from cortico-hippocampal regions from three mice per group were taken for Western blot analysis. Movie S1. Representative 3D reconstructions from SBFSEM of dendrites (yellow), mitochondria (red), and ER (blue) in hippocampi from (a) NTG, (b) APP/PS1, and (c) APP/PS1+CP2 mice. Movie S2. Representative 3D reconstructions highlighting MERCS (blue) in (a) NTG, (b) APP/PS1, and (c) APP/PS1 + CP2 mice. Table S1. Transcriptomic changes in cortico-hippocampal brain tissue from vehicle- and CP2-treated NTG and APP/PS1 mice were determined using RNA-seq. Differential expression analysis results and normalized expression of genes significantly changed and related to ER-stress, UPR, and mitochondrial-ER tethering were presented as log2-fold change, *p*-values, and FDR-adjusted *p*-values, between APP/PS1 and NTG, and between CP2- and vehicle-treated APP/PS1 mice.
