# Supplementary material for: Partial Inhibition of Complex I Restores Mitochondrial Morphology and Mitochondria-ER Communication in Hippocampus of APP/PS1 Mice
Source: Cells. 2023 Apr 8;12(8):1111. doi: 10.3390/cells12081111 (PMC10137328; doi:10.3390/cells12081111)
Supplement: Supplementary file 1 [file cells-12-01111-s001.zip › Figure S1 040723.pptx]

## Slide 1
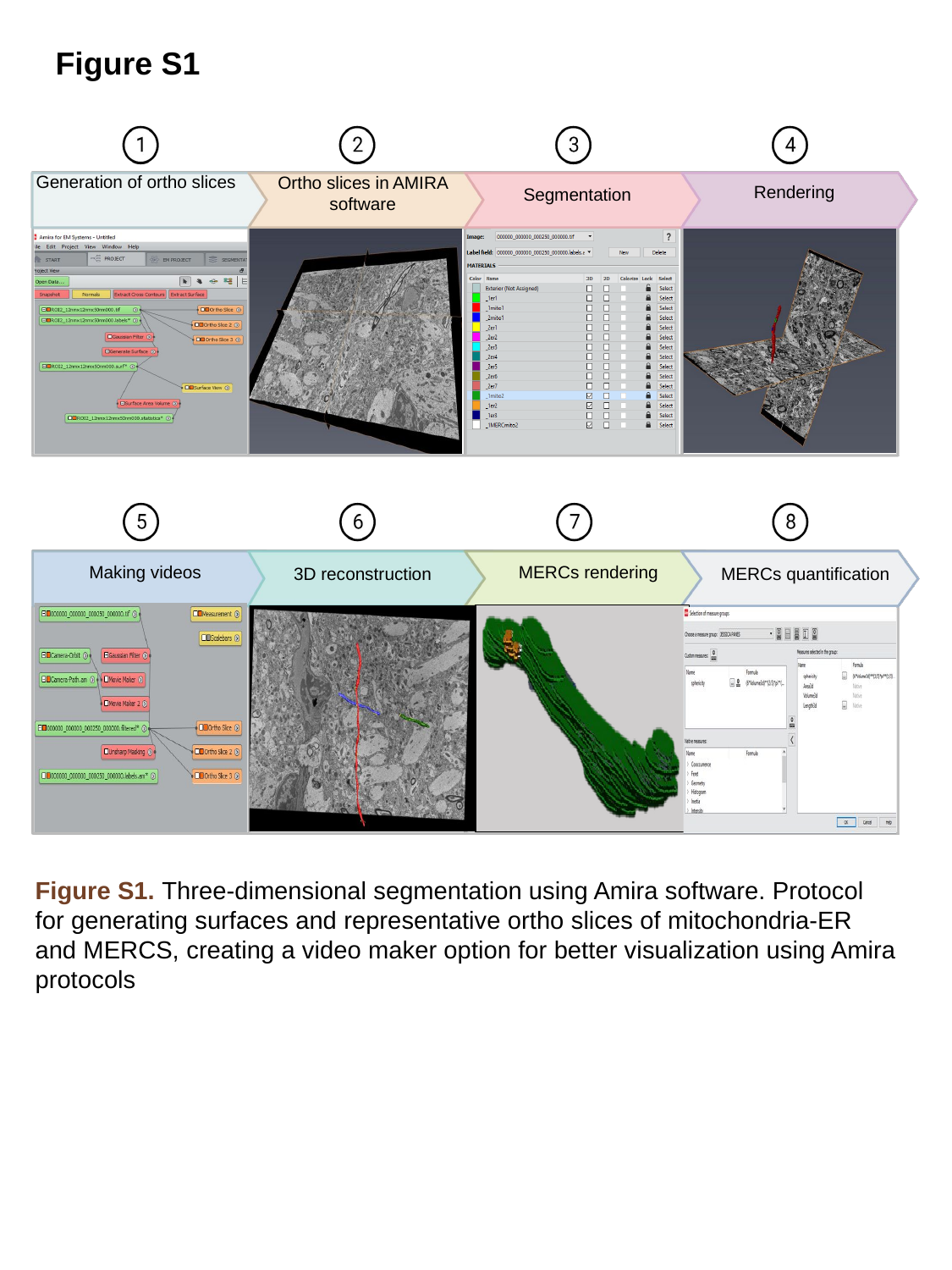

Figure S1
Generation of ortho slices
Ortho slices in AMIRA software
Rendering
Segmentation
MERCs rendering
Making videos
MERCs quantification
3D reconstruction
Figure S1. Three-dimensional segmentation using Amira software. Protocol for generating surfaces and representative ortho slices of mitochondria-ER and MERCS, creating a video maker option for better visualization using Amira protocols
