# Supplementary material for: Partial Inhibition of Complex I Restores Mitochondrial Morphology and Mitochondria-ER Communication in Hippocampus of APP/PS1 Mice
Source: Cells. 2023 Apr 8;12(8):1111. doi: 10.3390/cells12081111 (PMC10137328; doi:10.3390/cells12081111)
Supplement: Supplementary file 1 [file cells-12-01111-s001.zip › Figure S2 040723.pptx]

## Slide 1
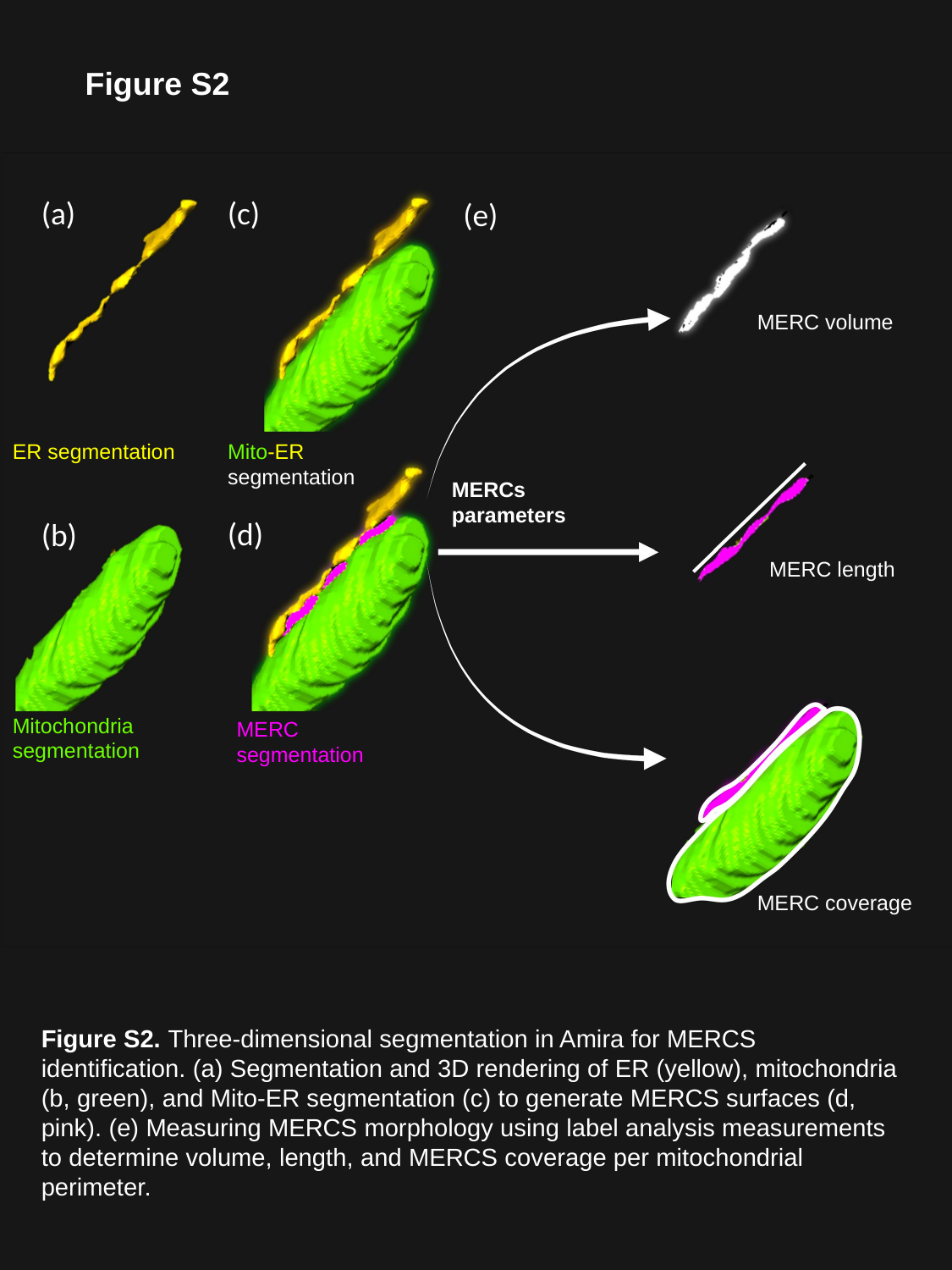

Figure S2
(a)
(c)
(e)
MERC volume
ER segmentation
Mito-ER segmentation
MERCs parameters
(d)
(b)
MERC length
Mitochondria segmentation
MERC segmentation
MERC coverage
Figure S2. Three-dimensional segmentation in Amira for MERCS identification. (a) Segmentation and 3D rendering of ER (yellow), mitochondria (b, green), and Mito-ER segmentation (c) to generate MERCS surfaces (d, pink). (e) Measuring MERCS morphology using label analysis measurements to determine volume, length, and MERCS coverage per mitochondrial perimeter.
