# Supplementary material for: Partial Inhibition of Complex I Restores Mitochondrial Morphology and Mitochondria-ER Communication in Hippocampus of APP/PS1 Mice
Source: Cells. 2023 Apr 8;12(8):1111. doi: 10.3390/cells12081111 (PMC10137328; doi:10.3390/cells12081111)
Supplement: Supplementary file 1 [file cells-12-01111-s001.zip › Figure S3 040723.pptx]

## Slide 1
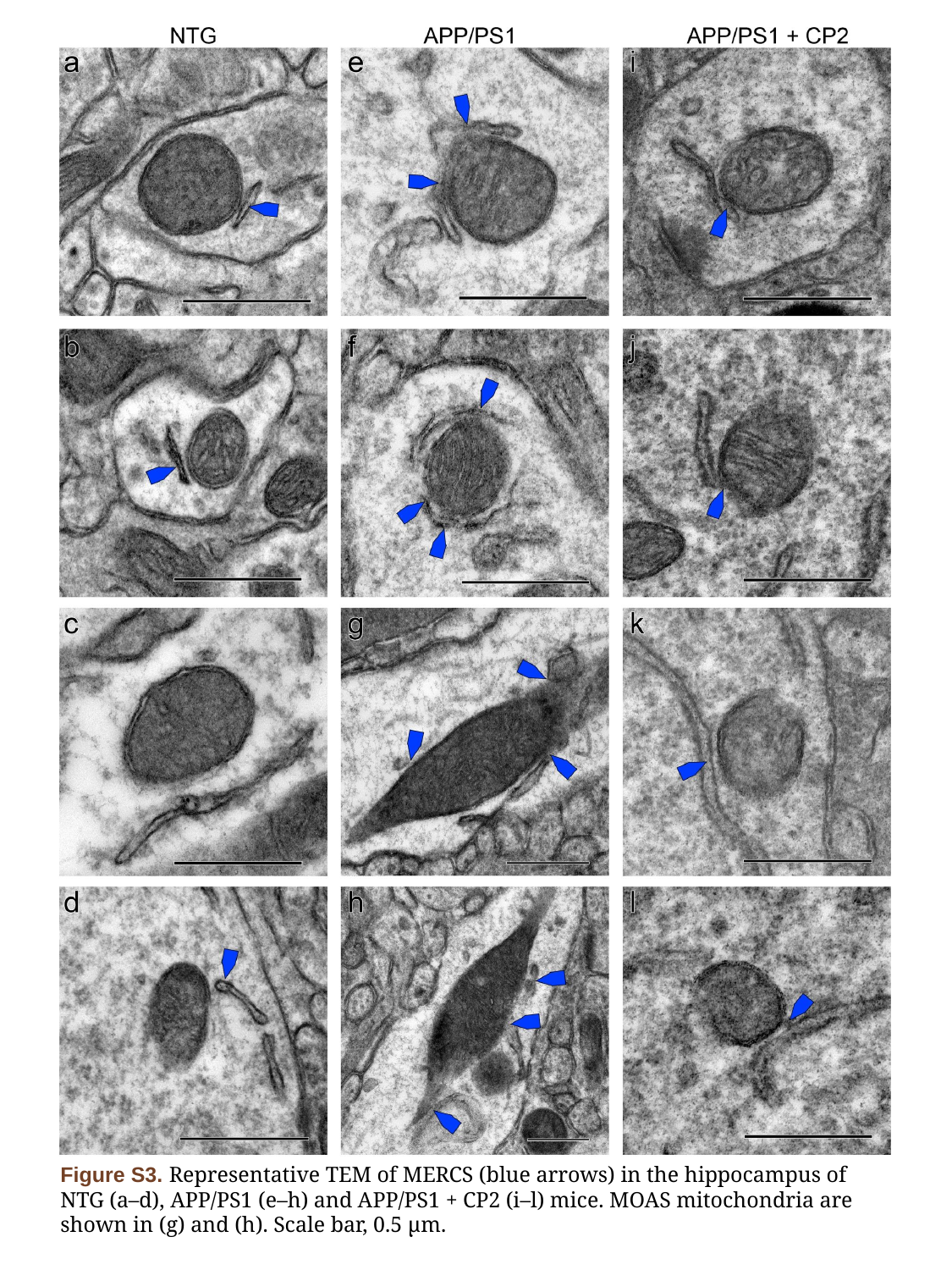

Figure S3. Representative TEM of MERCS (blue arrows) in the hippocampus of NTG (a–d), APP/PS1 (e–h) and APP/PS1 + CP2 (i–l) mice. MOAS mitochondria are shown in (g) and (h). Scale bar, 0.5 μm.
