# Supplementary material for: Partial Inhibition of Complex I Restores Mitochondrial Morphology and Mitochondria-ER Communication in Hippocampus of APP/PS1 Mice
Source: Cells. 2023 Apr 8;12(8):1111. doi: 10.3390/cells12081111 (PMC10137328; doi:10.3390/cells12081111)
Supplement: Supplementary file 1 [file cells-12-01111-s001.zip › Figure S4 040723.pptx]

## Slide 1
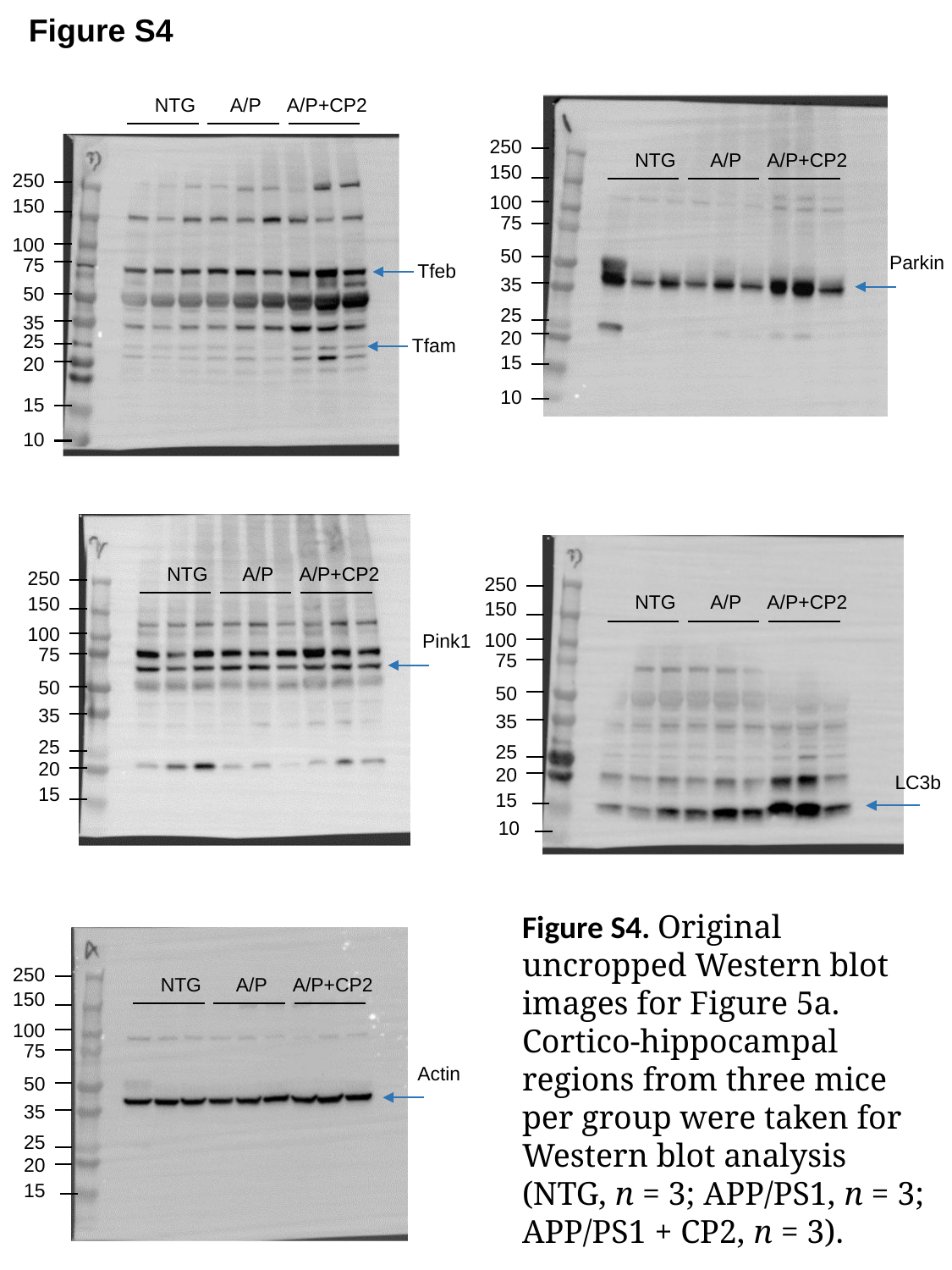

Figure S4
NTG
A/P
A/P+CP2
250
150
100
75
Tfeb
50
35
25
Tfam
20
15
10
250
NTG
A/P
A/P+CP2
150
100
75
50
Parkin
35
25
20
15
10
NTG
A/P
A/P+CP2
250
150
100
Pink1
75
50
35
25
20
15
NTG
A/P
A/P+CP2
LC3b
250
150
100
75
50
35
25
20
15
10
Figure S4. Original uncropped Western blot images for Figure 5a. Cortico-hippocampal regions from three mice per group were taken for Western blot analysis (NTG, n = 3; APP/PS1, n = 3; APP/PS1 + CP2, n = 3).
250
NTG
A/P
A/P+CP2
150
100
75
Actin
50
35
25
20
15
