# Supplementary material for: Partial Inhibition of Complex I Restores Mitochondrial Morphology and Mitochondria-ER Communication in Hippocampus of APP/PS1 Mice
Source: Cells. 2023 Apr 8;12(8):1111. doi: 10.3390/cells12081111 (PMC10137328; doi:10.3390/cells12081111)
Supplement: Supplementary file 1 [file cells-12-01111-s001.zip › Figure S5 040723.pptx]

## Slide 1
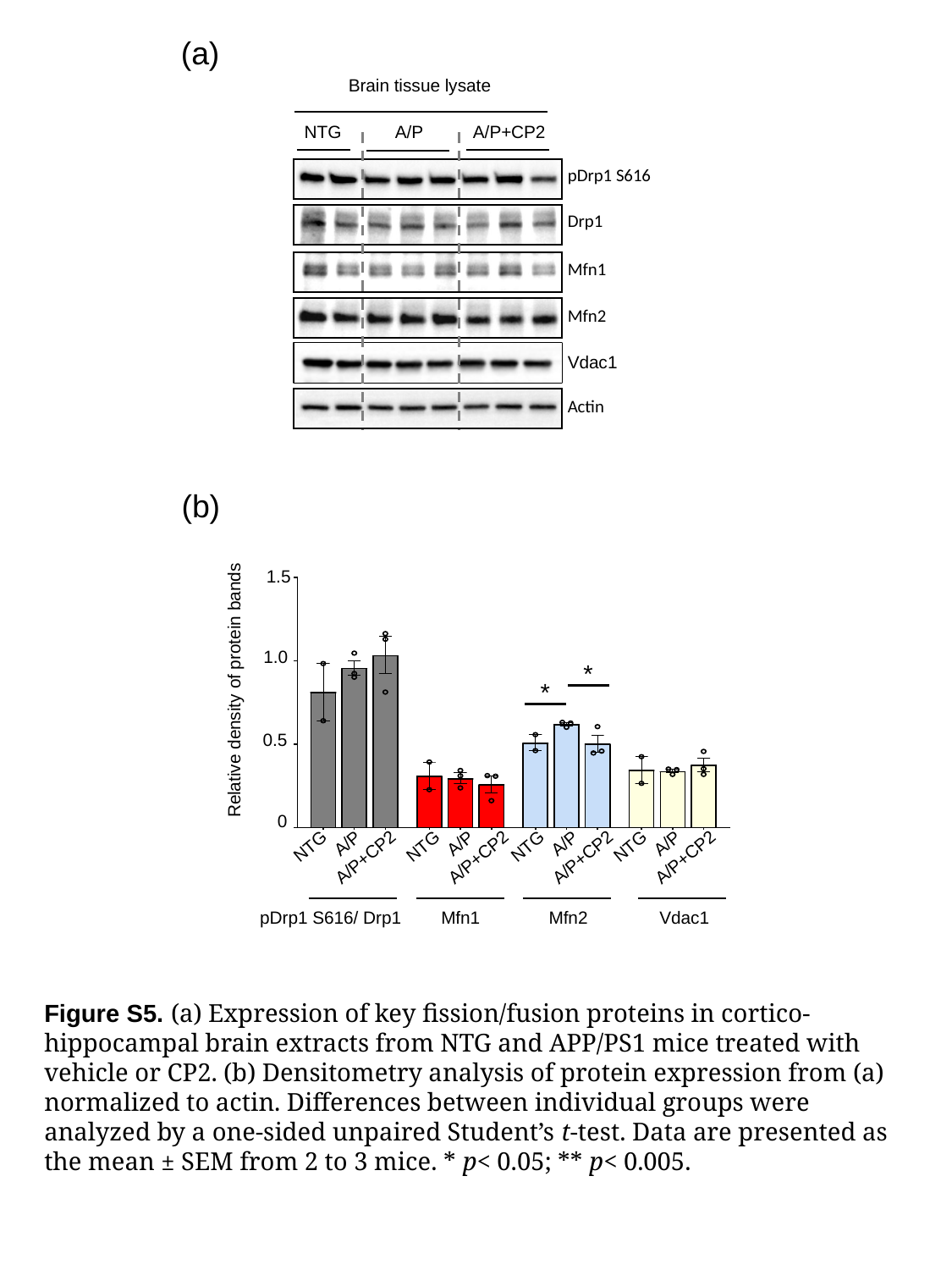

(a)
Brain tissue lysate
NTG
A/P
A/P+CP2
pDrp1 S616
Drp1
Mfn1
Mfn2
Vdac1
Actin
(b)
1.5
1.0
*
*
Relative density of protein bands
0.5
0
NTG
A/P
NTG
A/P
NTG
A/P
NTG
A/P
A/P+CP2
A/P+CP2
A/P+CP2
A/P+CP2
pDrp1 S616/ Drp1
Mfn1
Mfn2
Vdac1
Figure S5. (a) Expression of key fission/fusion proteins in cortico-hippocampal brain extracts from NTG and APP/PS1 mice treated with vehicle or CP2. (b) Densitometry analysis of protein expression from (a) normalized to actin. Differences between individual groups were analyzed by a one-sided unpaired Student’s t-test. Data are presented as the mean ± SEM from 2 to 3 mice. * p< 0.05; ** p< 0.005.
