# Supplementary material for: Partial Inhibition of Complex I Restores Mitochondrial Morphology and Mitochondria-ER Communication in Hippocampus of APP/PS1 Mice
Source: Cells. 2023 Apr 8;12(8):1111. doi: 10.3390/cells12081111 (PMC10137328; doi:10.3390/cells12081111)
Supplement: Supplementary file 1 [file cells-12-01111-s001.zip › Figure S6 040723.pptx]

## Slide 1
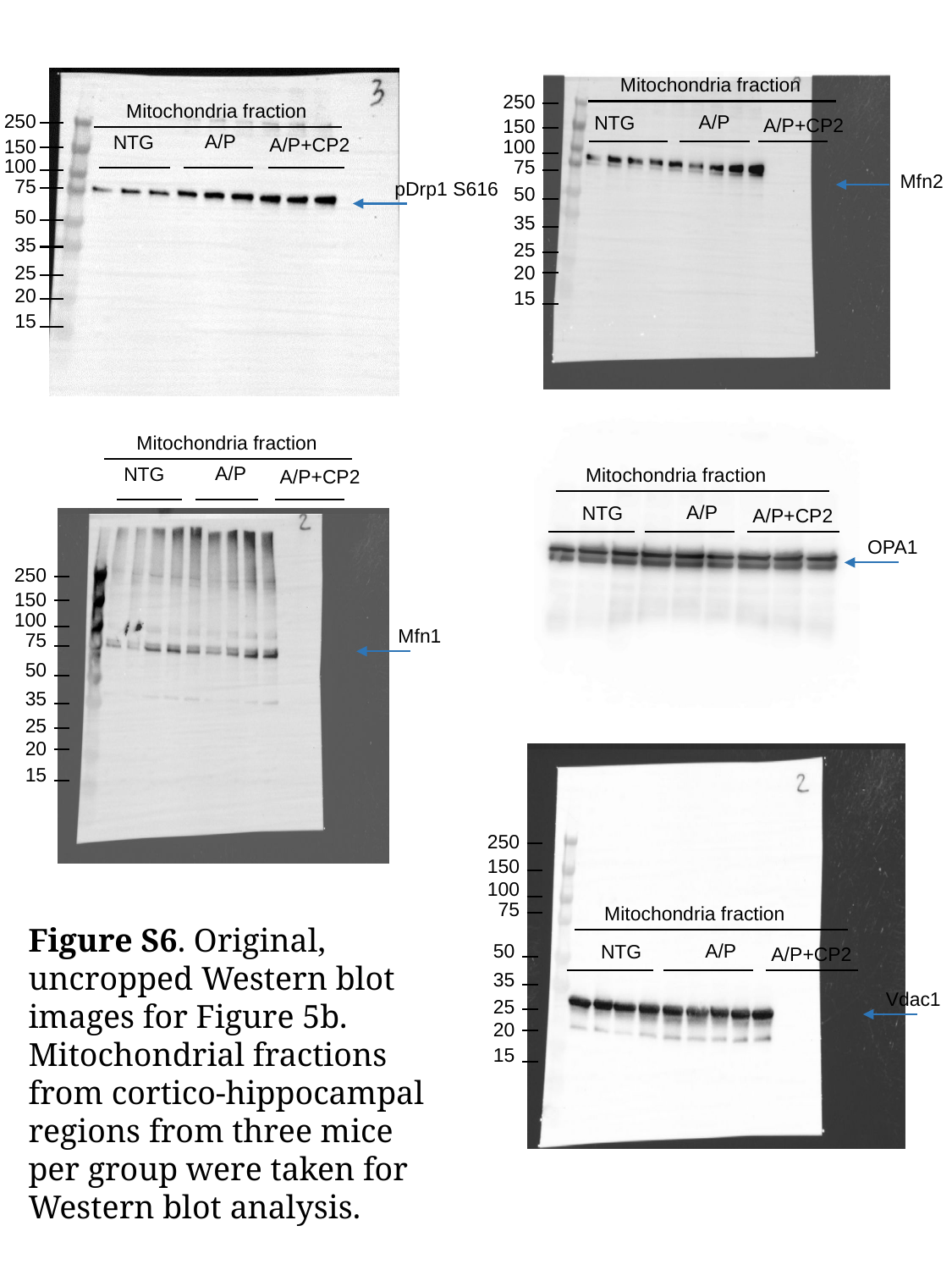

Mitochondria fraction
250
A/P
NTG
A/P+CP2
150
100
75
Mfn2
50
35
25
20
15
Mitochondria fraction
250
A/P
NTG
A/P+CP2
150
100
75
pDrp1 S616
50
35
25
20
15
Mitochondria fraction
A/P
NTG
A/P+CP2
OPA1
Mitochondria fraction
A/P
NTG
A/P+CP2
250
150
100
Mfn1
75
50
35
25
20
15
250
150
100
75
Mitochondria fraction
A/P
50
NTG
A/P+CP2
35
Vdac1
25
20
15
Figure S6. Original, uncropped Western blot images for Figure 5b. Mitochondrial fractions from cortico-hippocampal regions from three mice per group were taken for Western blot analysis.
